# A Unique Indication for the Altura Endograft to Repair Bilateral Common Iliac Artery Aneurysms Associated with a Short–Infrarenal Aorta

**DOI:** 10.1055/s-0040-1702142

**Published:** 2020-07-31

**Authors:** Stavros K. Kakkos, Chrysanthi P. Papageorgopoulou, Konstantinos Katsanos, Peter Zampakis, Athina Siampalioti, Ioannis Ntouvas, Chrysanthi-Helen Loizou, Eleni Tsamantioti, Spyros Papadoulas, Konstantinos M. Nikolakopoulos, Anastasia Kouri

**Affiliations:** 1 Department of Vascular Surgery, University of Patras Medical School, Patras, Greece; 2 Department of Radiology, University of Patras Medical School, Patras, Greece; 3 Department of Anesthesiology and Intensive Care, University of Patras Medical School, Patras, Greece

**Keywords:** iliac artery aneurysm, endograft, embolization

## Abstract

A 71-year-old man with end-stage renal disease on hemodialysis presented with bilateral common iliac artery aneurysms diagnosed during the workup of his chronic kidney disease. On computed tomography angiography, common iliac artery aneurysm diameters measured 6.1 cm on the right side and 3.1 cm on the left side. The infrarenal aorta also had a small 3.2-cm aneurysm, but the length from the lowest left renal to the aortic bifurcation was only 6.7 cm, precluding use of most bifurcated endografts. Following an uneventful staged preoperative internal iliac artery embolization, a two-piece **D** -shaped Altura endograft for the aorta, with bilateral iliac components, landing at the level of the external iliac arteries was successfully performed. Postoperative course was uneventful with no endoleak or endograft migration on computerized tomographic angiography 45 days later, although billowing mimicking an endoleak was evident and will be closely followed.

## Introduction

Endovascular aneurysm repair (EVAR) is currently the most commonly performed procedure to treat aortoiliac aneurysms, surpassing open repair a long time ago. Restricted by certain anatomic factors of the aneurysm, EVAR technology is, however, continuously evolving and expanding our armamentarium. Low-profile delivery systems, fenestrated endografts, endoanchors, and endovascular aneurysm sealing methodologies, 1 2 3 among others, are often used in challenging anatomies. This is particularly crucial for high-risk patients including those with respiratory, heart, or chronic kidney disease (CKD). Herein, we present the case of a patient with bilateral common iliac artery aneurysms (IAAs) associated with a short–infrarenal aorta that constituted a contraindication to most conventional endografts. The patient was successfully managed with the low-profile Altura endograft system, following embolization of the internal iliac arteries.

## Case Presentation

A 71-year-old man with end-stage renal disease on hemodialysis was referred to our outpatient clinic with bilateral common IAAs, incidental findings during the workup to rule out obstructive uropathy or a renal parenchymal disease as potential causes of his CKD. He had known CKD for over a year, and 3 months earlier he started a regular hemodialysis program at an outside facility, currently through a right internal jugular vein catheter. Past medical history besides CKD included coronary artery disease with percutaneous transluminal coronary angioplasty of one vessel, about 8 years ago, hypertension, dyslipidemia, depression, and glaucoma. Orally taken medications included aspirin 100 mg QD, clopidogrel 75 mg QD, diltriazem 60 mg TID, carvedilol 3.125 mg BID, clonidine 150 mg (1–1/2–1), atorvastatin 20 mg QD, duloxetide 60 mg QD, and omeprazole 20 mg QD. Additionally, he was on latanoprost eye drops QD. There were no known allergies. Past surgical history included open cholecystectomy, 10 years ago, and more recently excision of a basal cell skin carcinoma with part of the underlying mandible in his left zygomatic region. He had never been a smoker. Family history was negative for aortic or other aneurysms. On physical examination of the abdomen, a scar from a right Kocher's incision, and also obesity was noted, with no palpable masses. A right internal jugular vein hemodialysis catheter was noted. There was mild peripheral edema in his legs, and femoral and dorsalis pedis pulses were normal. We did not palpate popliteal artery aneurysms. A scar in his left zygomatic region was noted. No carotid bruits were auscultated. The remaining examination was unremarkable.

**Fig. 1:**
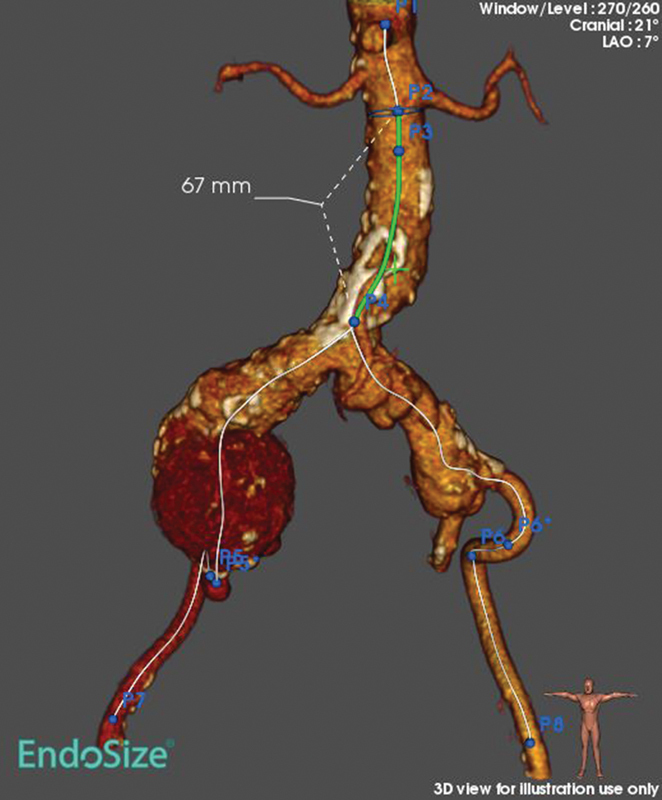
Three-dimensional reconstruction computed tomography image (arterial phase) of the abdominal aorta and the iliac arteries. The presence of bilateral iliac artery aneurysms and a short infrarenal portion of the aorta can be noted. LAO, left anterior oblique.

On computerized tomographic (CT) angiography ( Fig. 1 ), common IAAs were noted, with diameters measuring 6.1 cm on the right side and 3.1 cm on the left side. The infrarenal aorta showed also a small 3.2-cm aneurysm. Aortic length from the lowest left renal to its bifurcation was only 6.7 cm. The infrarenal neck was straight, with a diameter of 19.6 mm below the lowest renal, 27 mm in length, and an infrarenal angle of 16.2 degrees. Diameter of external iliac arteries was at least 7.8 mm. Celiac artery and superior and inferior mesenteric arteries were patent with no stenotic lesions being detected.

Preoperative workup was performed to identify an endovascular solution for this apparently high-risk patient with coronary artery and chronic kidney disease. Conventional endografts were not suitable because of the short length of the infrarenal aorta, as described above, which was shorter than the shortest commercially available main body of all but one endograft available on the market.

An Altura endograft (Altura Endograft System, Lombard Medical, Didcot, Oxfordshire, United Kingdom), which is polyester with a nitinol frame featuring active suprarenal fixation and double **D** -shaped proximal stents) was suitable. However, there was no adequate landing zone in the common iliac arteries bilaterally. As part of the preoperative preparation, staged preoperative IAA embolization was decided. Staging was opted to reduce the possibility of pelvic floor and sigmoid colon ischemia, likely to be possible based on patient's past medical history and a patent inferior mesenteric artery, respectively. Initially, embolization of the left IAA with a 16-mm Amplatzer-II vascular plug (St. Jude Medical, MN) delivered with a crossover technique from the contralateral side, through a standard 6-Fr vascular sheath, was performed on an outpatient basis. Vessel occlusion was confirmed after a few minutes angiographically. Approximately a month later, our patient was admitted and underwent embolization of the right IAA with a 16-mm Amplatzer-II vascular plug (St. Jude Medical, MN) using the same technique, without any complication. Vessel occlusion was confirmed after a few minutes on completion angiography.

The next day, the Altura Endograft System, with its bilateral iliac components, was deployed, landing at the level of the external iliac arteries, with technical details as described below.

Through standard longitudinal groin incisions, the common femoral artery was identified, dissected and controlled with vessel loops, bilaterally. Introducer sheaths were exchanged for the delivery systems of the two aortic components of the stent graft (diameter, 24 mm; length, 90 cm; Altura Endograft System, Lombard Medical), one for each side, which were advanced to the level of the infrarenal aorta in a crossed limb configuration.

**Fig. 2:**
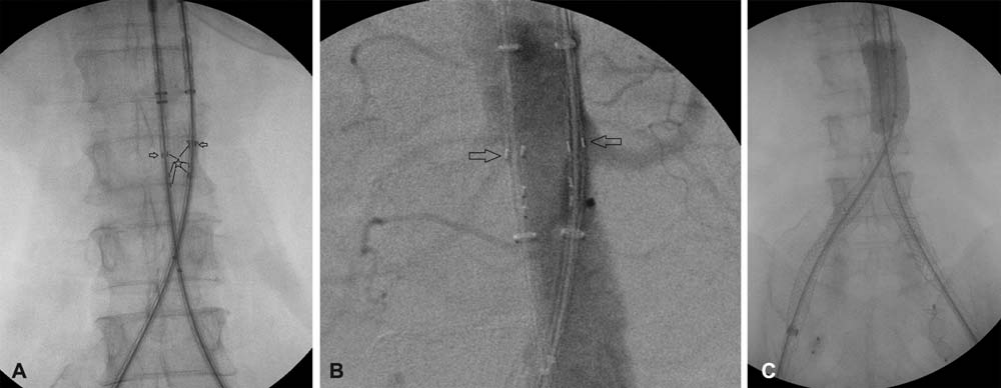
( **A** ) Intraoperative X-ray using the C-arm demonstrating the aortic components of the Altura stent graft appropriately positioned so that the three markers (star with lines) of each component are aligned medially opposite each other and the renal markers (arrows) are aligned laterally. ( **B** ) Intraoperative aortogram showing proper alignment of the aortic components, but somewhat cephalad compared with the renals, not allowing deployment before caudal movement. ( **C** ) Kissing balloon technique with two 32 mm CODA (COOK MEDICAL) molding balloon catheters dilating the proximal parts of the aortic components of the stent grafts.

We proceeded with the proper alignment of the stent grafts by rotating the delivery system so that three radiopaque tantalum markers on both stent grafts aligned with each other, allowing the flat faces of the two **D** -shaped stent grafts (unique for the aortic components) to be aligned in a medial position ( Fig. 2A ), to ensure proper positioning of this modular endograft and prevent endoleak. After the alignment, we rotated the handle on one of the delivery systems to unsheathe the proximal end of the stent graft, so that we could perform an angiography through the delivery system, and this way we identified the renal arteries, with the left one being the lowest one. The single lateral marker of each aortic component was used to position them just below the renal arteries ( Fig. 2B ). We then rotated the handle on both devices to expose the proximal end of both stent grafts, with further adjustments being made to properly orientate the grafts. We then proceeded with the compression maneuver of the proximal end of the stent grafts, using the mechanism on the delivery system, to form the **D** -shape on the proximal end. Because one of the devices was not properly aligned, the mechanism used to form the **D** -shape was reversed allowing accurate repositioning and adjustment of the alignment of the stent-graft.

Subsequently, we proceeded with releasing the proximal bare stent, on both devices, which has the fixation barbs, and then finished deploying the aortic components. Since this graft does not require cannulation, we proceeded with placement of the device for right iliac component (diameter, 13 mm and length, 65 cm, Altura Endograft System, Lombard Medical) and then a similar left iliac device over Lunderquist wires (COOK MEDICAL, Bloomington, IN). These stent-grafts deploy from the distal end to the proximal end allowing for proper placement at the common iliac bifurcation, although on this occasion, the distal landing zones were the external iliac arteries, bilaterally. The delivery systems of the two stent grafts were replaced with two 16-Fr 30-cm introducer sheaths (COOK MEDICAL). Two 32-mm CODA (COOK MEDICAL) molding balloon catheters were used to dilate the stent grafts, particularly at the neck using the kissing balloon technique ( Fig. 2C ) and their joint areas.

**Fig. 3:**
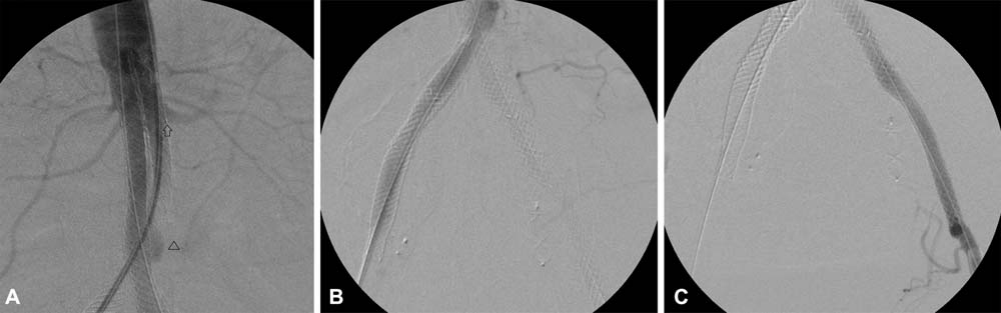
Completion aortogram showing proper placement of all stent graft components (aortic components in **A** , indicated by an arrow, and iliac limbs in **B** and **C** ) with no endoleak. However, a possible bulging was visualized in the right aortic component, corresponding to the left iliac limb due to crossed limb configuration (arrowhead, **A** ).

A completion aortogram through an 8-Fr 45-cm introducer sheath (Cordis) placed through the right common femoral showed proper placement of all stent-graft components and no endoleaks ( Fig. 3 ), although a possible bulging was visualized in the right aortic component, corresponding to the left iliac limb due to crossed limb configuration (arrowhead, Fig. 3A ). Fluroscopy time was 28 minutes and contrast material totaled 140 mL. Peripheral pulses were present at the conclusion of the procedure. The postoperative course was uneventful, without any complications.

The patient was discharged home on the second postoperative day.

**Fig. 4:**
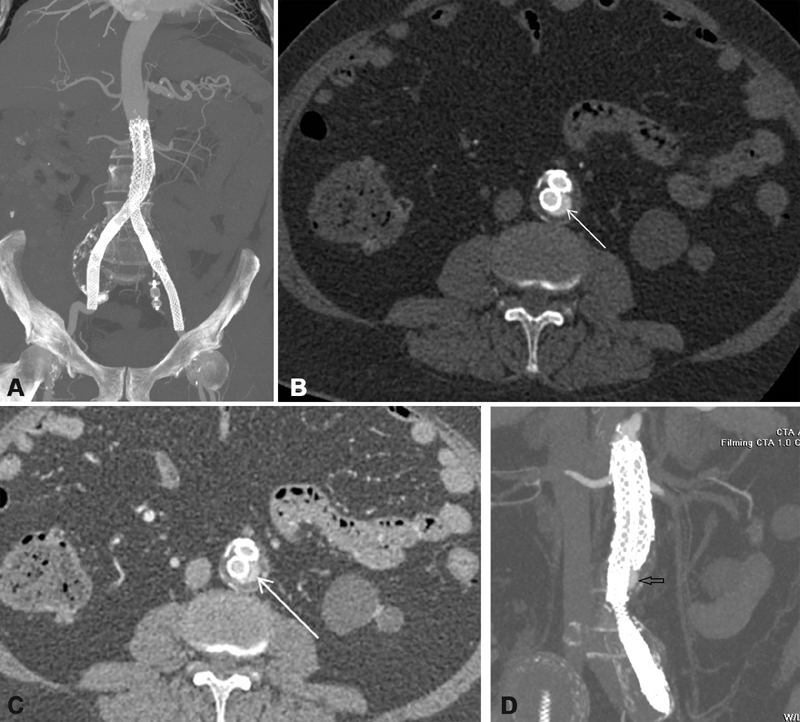
( **A** ) Computed tomography angiography of the abdominal aorta at 45 days after the procedure. Maximum intensity projection (MIP) coronal reconstruction shows excellent patency of the aortic graft. ( **B** and **C** ) Axial images of the aorta at the level of L3 vertebrae. On the arterial phase ( **B** ), the white arrow indicates protrusion of the contrast contour of the right aortic component and on the venous phase ( **C** ) contrast protrusion has the same appearance with arterial phase (white arrow). ( **D** ) This is indicative of billowing of the right aortic component, which continues as the left iliac component and is further evident (arrow) in the three-dimensional MIP reconstruction.

Repeat imaging with CT angiography, 45 days later, ruled out endoleak or endograft migration; however, billowing of one of the aortic components mimicking an endoleak was evident ( Fig. 4 ) at the point bulging was seen intraoperatively ( Fig. 3A ). Aneurysm sacs were slightly reduced to 5.8 and 2.9 cm for the right and left IAAs, respectively. On further imaging with color-coded duplex ultrasonography on the sixth postoperative month, there was no endoleak detected, and aneurysm sacs were further reduced to 5.3 and 2.7 cm for the right and left IAAs, respectively. Our patient is closely followed-up with repeat imaging planned for the 12th postoperative month.

## Discussion

We present the case of a 71-year-old man with end-stage renal disease on hemodialysis with bilateral common IAAs and a short–infrarenal aorta that precluded use of most conventional endografts. These anatomic issues were successfully managed with a two-piece **D** -shaped Altura endograft for the aorta with bilateral iliac components landing at the level of the external iliac arteries.

A short–infrarenal aorta presents a special challenge for most endograft types because their main body is made by the various manufacturers to be longer than the aorta; this cut-off point varies among the various endograft types. Actually, it is the length between the proximal part of the body and the contralateral gate that restricts its use in infrarenal aortic segments of a certain length, so that the contralateral gate opens inside the aorta and not the ipsilateral iliac artery, thus preventing cannulation from the contralateral side and inline placement of the contralateral limb.

In our case, the AFX endovascular AAA system (Endologix, Irvine, CA) would have been an alternative solution, because its main body is made as short as 40 mm in length. Similarly, the Nellix Endovascular Aneurysm Sealing System (EVAS; Endologix) could have been used, although currently available only within clinical trials. Placement of an endograft with bilateral limbs, such as in our case obviated use of an aortouniiliac endograft, contralateral iliac occlusion and femorofemoral bypass grafting, known to be associated with a higher risk of infection than totally endovascular solutions.

Our patient was at a high risk for open repair mainly because of his history of end-stage renal disease requiring hemodialysis. Even EVAR in this patient population carries substantial risks, with mortality figures up to 5.2% in one recent study. 4 Given that this risk is not prohibitive for EVAR, we proceeded after bilateral hypogastric embolization. Still the later procedure in combination with cessation of flow to the inferior mesenteric artery following EVAR may be associated with pelvic and sigmoid colon ischemia, particularly in this morbid patient population. However, such complications were not observed in our case and were thought to occur less often than a fatal outcome following open repair or expectant management.

We successfully implanted an Altura endograft with no evidence of complications on CT angiography a month and a half later. Published experience with this endograft type is excellent. 5 6 In a single series on 89 patients, endografts were successfully implanted in 99%. 5 Despite a 4.5% reintervention rate during the first 30 days, on midterm follow-up (median, 12.5 months; range, 11.5–50.9), there were no aneurysm ruptures, surgical conversions, or AAA-related deaths. Clinical success was reported to be 94% (84/89) at 30 days, 98% (85/87) at 6 months, and 99% (82/83) at 1 year. The authors concluded that properly selected AAA patients can be safely treated using the Altura Endograft System with favorable midterm outcome.

No endoleak or endograft migration on CT angiography was seen in our case at 45-day follow-up, indicating excellent technical and clinical success. Billowing mimicking an endoleak was, however, evident on imaging. This represents aneurysmal degeneration of the outer fabric material manifesting as a bulging sac of contained contrast due to weakening of the fabric material between the metal struts, which are not attached to the fabric material except at the two ends. Although contrast material is manifest beyond the skeleton, it is still contained within the graft. Although billowing is considered a benign finding, 7 recent reports have cast some doubt. 8 9 Billowing has been reported only for the Powerlink endograft so far, 7 8 9 10 and we believe that this is the first reported case of billowing in a patient with the Altura endograft. The lack of long-term data on this graft (median, 12.5 months) should be viewed as a limitation to its use. We will closely monitor our patient.
